# SNP Analysis of Caries and Initial Caries in Finnish Adolescents

**DOI:** 10.1155/2018/1586762

**Published:** 2018-04-23

**Authors:** Teija Raivisto, AnnaMaria Heikkinen, Leena Kovanen, Hellevi Ruokonen, Kaisa Kettunen, Taina Tervahartiala, Jari Haukka, Timo Sorsa

**Affiliations:** ^1^Department of Oral and Maxillofacial Diseases, University of Helsinki and Helsinki University Hospital, Helsinki, Finland; ^2^Department of Health, National Institute for Health and Welfare, Helsinki, Finland; ^3^Institute for Molecular Medicine Finland (FIMM), Helsinki, Finland; ^4^Department of Public Health and Clinicum, University of Helsinki, Helsinki, Finland; ^5^Department of Dental Medicine, Karolinska Institutet, Huddinge, Sweden

## Abstract

**Background:**

Dental caries is the most common infection in the world and is influenced by genetic and environmental factors. Environmental factors are largely known, but the role of genetic factors is quite unknown. The aim was to investigate the genetic background of caries in Finnish adolescents.

**Materials and Methods:**

This study was carried out at the Kotka Health Center in Eastern Finland. 94 participants aged 15–17 years gave approval for the saliva and DNA analyses. However, one was excluded in DNA analysis; thus, the overall number of participants in analysis was 93. Caries status was recorded clinically and from bite-wing X-rays to all 94 participants. Genomic DNA was extracted by genomic QIAamp® DNA Blood Mini Kit and genotyped for polymorphisms. The results were analyzed using additive and logistic regression models.

**Results:**

No significant associations between caries and the genes studied were found. However, SNPs in *DDX39B* and *MPO* showed association tendencies but were not statistically significant after false discovery rate (FDR) analysis. SNPs in *VDR*, *LTA*, and *MMP3* were not statistically significant with initial caries lesions after FDR analysis.

**Conclusion:**

The present study could not demonstrate statistically significant associations between caries and the genes studied. Further studies with larger populations are needed.

## 1. Introduction

Dental caries is a common chronic biofilm infection. *Streptococcus mutans* is the primary agent of dental caries; however, its role as a primary pathogen appears less pronounced in populations with prevention programs. In Swedish population, caries-active adolescents were colonized by *Actinomyces*, *Selenomonas*, *Prevotella*, and *Capnocytophaga* species [1]. Environmental and socioeconomic factors, reduced salivary flow, tooth structure, and oral dietary and hygienic habits also enhance the caries development and progression. However, there is only a spare information about the effects of genetic background of caries on adolescents [2].

*DDX39B* (BAT1) belongs to the DEAD-box family of RNA-binding proteins and is encoded in the central human major histocompatibility complex (MHC), which contains numerous genes involved in immune and inflammatory responses. The human leukocyte antigen- (HLA-B-) associated BAT1 is an RNA helicase encoded by the *DDX39B* gene (*BAT1*). BAT1 is shown to be a negative regulator of inflammation [3]. *DDX39B* encodes an RNA helicase known to regulate the expression of two cytokines, such as tumor necrosis factor-alpha (TNF-*α*) and interleukin-6 (IL-6) [4]. These both cytokines are well known in inflammatory areas. IL-6 acts in both proinflammatory and anti-inflammatory ways, and TNF-*α* is a monocyte-derived cytotoxin. BAT1 (*D6S81E* and *UAP56*) lies in the central MHC between *TNF* and *HLA-B*, a region containing genes that affect susceptibility to immunopathologic disorders. BAT1 protein may be directly responsible for the genetic association, as antisense studies show that it can downregulate inflammatory cytokines [5].

Myeloperoxidase (MPO) is a peroxidase enzyme. It is most abundantly expressed in neutrophil granulocytes in saliva and is a strong biomarker for both acute and chronic inflammatory conditions. Its main role is to produce hypochlorous acid to carry out the antimicrobial activity [6]. MPO also oxidatively activates latent forms of matrix metalloproteinases (MMPs), especially MMP-8 and MMP-9, and inactivates tissue inhibitors of metalloproteinases (TIMPs) and serpins [7–10].

Lactoferrin is present in or on all mucosal surfaces throughout the body and specifically in saliva. It can act as a host defense protein against *Streptococcus* species operating in the innate arm of the immune system, but also affecting adaptive immunity [11, 12]. Human lactoferrin (HLF) prophylaxis significantly decreased the expression levels of interferon gamma (IFN-*γ*), TNF-*α*, interleukin-1 beta (IL-1*β*), IL-6, MPO, and nitric oxide synthases (iNOS) [13]. IFN-*γ* is a cytokine participating in the immune system. IL-1*β* is also a cytokine present in humans encoded by the *IL1B* gene. The inducible isoform of iNOS is involved in immune response and inflammatory processes.

The aim of this study was to investigate the genetic background related to caries lesions in Finnish adolescents.

## 2. Materials and Methods

This study was carried out at the Kotka Health Center in Eastern Finland. The sample was collected in 2004-2005 and 2014-2015, comprising 15- to 17-year-old adolescents. Every 15- to 17-year-old living in Kotka was invited to examination in 2004-2005. In 2014-2015, 15- to 17-year-olds were invited to examination according to their individual examination time. The study flow of the participants is described by Heikkinen et al. [14]. This study was approved by the Ethical Committee of the Kymenlaakso Regional Hospital and by the Ethical Committee of the Helsinki and Uusimaa Hospital District (Dnro 260/13/03/00/13). The participants gave written informed consent. Altogether, there were 94 participants who gave their approval for the saliva and DNA analyses. However, in DNA analysis, one was excluded because of discrepant gender check; thus, the overall number of participants in analysis was 93. Caries status (*D* = at least one caries lesion in permanent dentition), including initial caries lesions, was recorded clinically and from bite-wing X-rays as well as visible plaque index (VPI) and smoking habits (current smoker/former smoker/nonsmoker) to all adolescents (*n*=94).

The selection process of single-nucleotide polymorphisms (SNPs) from caries-related candidate genes, DNA extraction, genotyping, and genotyping quality control have been described previously [14]. Genotyping success rates ranged from 90.3 to 98.9%. One subject was excluded due to discrepant gender check results.

Genomic DNA was extracted from 300 *μ*l of the saliva samples by genomic QIAamp DNA Blood Mini Kit (Qiagen, Hilden, Germany) and genotyped for polymorphisms. Genetic variants for genotyping were selected from the following genes of interest: *S100A8*, *FCGR2A*, *FCGR2B*, *IL10*, *MMP8*, *MMP3*, *MMP13*, *VDR*, *TLR4*, *MMP2*, *MPO*, *ELANE*, *IL1A*, *IL1B*, *IL1RN*, *CD28*, *MMP9*, *DDX39B*, *NFKBIL1*, *LTA*, *TNF*, *SOD2*, *IL6*, *TLR4*, *TIMP1*, and *SYN1*.

A logistic regression model was used to model the association between dichotomic outcome variables (decayed and initial caries) and 63 SNPs in the 93 subjects. An additive effect of SNPs was assumed. For both outcomes, two models were calculated: an unadjusted model with SNP as the only explanatory variable and an adjusted model with VPI, smoking (weekly smoking), and study period (2004-2005/2014-2015). *p* values for each outcome were corrected for multiple testing using the false discovery rate (FDR) [15, 16]. The significance level was set to FDR *q* value less than 0.05. All data analyses were carried out using R software version 3.2.2. [17] and SNPassoc package [18].

## 3. Results

Based on our caries status, of the participants, 30 had at least one caries lesion, 57 did not have any caries lesion, and data were missing from seven patients. Sixty participants had at least one initial caries lesion, 28 did not have any initial caries lesion, and data were missing from six patients. Of all participants, 16 were smoking regularly and 77 were nonsmokers. From one patient, information concerning smoking was missing. In DNA analysis, one was excluded because of discrepant gender check; thus, the final number of participants in analysis was 93. General characteristics of the participants are represented in Table 1. SNPs in *DDX39B* (rs7766569, *p*=0.03, *q*=0.688885) and *MPO* (rs2243828, *p*=0.04, *q*=0.688885) showed association tendencies, which however remained not statistically significant when corrected for multiple testing by the false discovery rate (FDR). Other studied SNPs did not reveal any associations (Table 2). SNPs in *VDR* (rs2228570, *p*=0.01, *q*=0.50), *LTA* (rs2009658, *p*=0.02, *q*=0.50), and *MMP3* (rs650108, *p*=0.03, *q*=0.61) showed no statistically significant associations after FDR analysis with initial caries lesions (Table 3).

## 4. Discussion

Dental caries is a multifactorial disease caused by environmental factors and behavioral risk factors. These factors include diet, bacterial flora, oral self-care, salivary flow and composition, fluoride exposure, tooth morphology, and access to dental care. The role of genetic factors on caries risk is still largely unknown but has been investigated in several recent studies [2, 19, 20]. Therefore, we aimed to investigate the genetic background related to caries lesions in Finnish adolescents.

Following genes were studied: *S100A8*, *FCGR2A*, *FCGR2B*, *IL10*, *MMP8*, *MMP3*, *MMP13*, *VDR*, *TLR4*, *MMP2*, *MPO*, *ELANE*, *IL1A*, *IL1B*, *IL1RN*, *CD28*, *MMP9*, *DDX39B*, *NFKBIL1*, *LTA*, *TNF*, *SOD2*, *IL6*, *TLR4*, *TIMP1*, and *SYN1*. In the present study, association tendencies between the studied SNPs in *DDX39B* (rs7766569, adjusted OR 3.39, 95% CI 1.10–10.43) and *MPO* (rs2243818, adjusted OR 2.33, 95% CI 0.97–5.58) and dental caries were found; however, statistical significance disappeared after correction for multiple testing (Tables 2 and 3). Furthermore, SNPs in *VDR* (rs2228570), *LTA* (rs2009659), and *MMP3* (rs650108) with initial caries lesions did not remain significant after FDR correction.

In a previous study, a *VDR-FokI* gene polymorphism was associated with dental caries in 12-year-old adolescents in China. Four *VDR* gene polymorphisms were examined, and the other three *VDR* gene polymorphisms (*Bsm* I, *Taq* I, and *Apa* I) showed no statistically significant differences in the caries group compared with the controls [21]. In a Czech study, the *VDR TaqI* gene variant could not be used as a marker for identification of children with increased dental caries risk either [22]. However, according to another study by Cogulu et al. [23], *VDR* gene polymorphisms may be used as a marker for the identification of patients with high caries risk [23].

Several studies have demonstrated that MMPs are involved in dental caries. The release of acids by bacteria rapidly decreases the pH in saliva, and the acidic environment can then activate host-derived pro-MMPs from both dentin and saliva [24]. The importance of MMPs in the development and progression of dentin caries has been studied by Tjäderhane et al. [25]. They found that human MMP-2, MMP-8, and MMP-9 were identified in demineralized dentinal lesions and inhibition of MMPs can reduce dentin caries progression. Host MMPs, activated by bacterial acids, may have a crucial role in the destruction of dentin by caries. *MMP9* and *MMP20* were involved in white spot lesions and early childhood caries development according to the study of Antunes et al. [26]. Lewis et al. [27] investigated SNPs in or near three *MMP* genes (*MMP10*, *MMP14*, and *MMP16*) for evidence of association with dental caries. Significant evidence of association was seen between two SNPs upstream of *MMP16* with dental caries [27]. The allele frequency of *MMP2*, *MMP13*, and *TIMP2* was different between caries-affected and caries-free individuals, with significant association for *MMP13* [28]. *MMP2* and *MMP3* genes are likely to be involved in caries [29]. However, we did not find any association between the studied *MMP2*, *MMP3*, *MMP8*, *MMP9*, and *MMP13* SNPs and caries.

The heritability of dental caries varies between 40% and 60% [2, 20, 30]. Wang et al. [31] observed in their studies that SNPs in three genes, namely, dentin sialophosphoprotein (*DSPP*), aquaporin 5 (*AQP5*), and kallikrein-related peptidase 4 (*KLK4*), showed consistent associations with protection against caries. Genes involved in enamel formation (*AMEX*, *AMBN*, *ENAM*, *TUFT*, *MMP20*, and *KLK4*), salivary characteristics (*AQP5*), immune regulation, and dietary preferences had the largest impact [27]. *DSPP* gene encodes two principal proteins of the dentin extracellular matrix of the tooth. *AQP5* encodes a series of homologous membrane proteins. *KLK4* is playing a role in enamel mineralization. Two genes, namely, taste 2 receptor member 38 (*TAS2R38*) and taste 1 receptor member (*TAS1R2*), have been identified to be important in taste sensing and to be associated with dental caries risk and/or protection. Dental caries is heritable, and genes affecting susceptibility to caries in the primary dentition may differ from those in permanent teeth [2]. This may help to identify the individuals at risk and enhance the implementation of preventive strategies before the onset of caries [20].

Smoking can be a confounding factor. Tobacco smoking has been found to increase the risk for dental caries [32]. The high rate of restorative treatment may also be explained by poor oral health behaviors [33, 34].

The sample size was quite small in this study of adolescents, and it needs to be acknowledged. Majority of the participants were lost because in Finland, it is difficult to obtain permission from adolescents for a genetic study.

## 5. Conclusion

In conclusion, no significant associations between SNPs in *DDX39B* and *MPO* genes and dental caries were found in the present study; however, a tendency could be observed, as well as between initial caries and polymorphisms in *VDR*, *LTA*, and *MMP3*. SNPs in other genes were also investigated, but none of these showed a significant relationship, not either for initial caries. Altogether, dental caries is influenced by genetic and environmental factors. Several genes are likely to have influence on dental caries. More studies with larger populations and sample sizes are needed for final conclusion.

## Figures and Tables

**Table 1:** General characteristics of the participants with nonmissing DNA sample (*n*=94).

Characteristics	*n*	%	Missing, *n*
*Gender*			
Male	47^a^	50.0	
Female	47^a^	50.0	

*Caries*			
Initial caries lesions	60	68.2	6
No initial caries lesions	28	31.8	
Caries lesions	30	34.5	
No caries lesions	57	65.5	7

*Smoking*			
Smoking regularly	16	17.2	1
Nonsmokers	77	82.8	

^a^One was excluded because of discrepant gender check; thus, *n*=93 the overall number of participants in DNA analysis was 93.

**Table 2:** Gene, chromosomal location, and SNPs associated with decayed (D) unadjusted and adjusted values and *p* values and FDR *q* values.

Gene	Chr. location	Marker	Allele	OR	Lower	Upper	*p* value	FDR *q* value
*Unadjusted values*								
*DDX39B*	2807359	RS7766569	*G*	2.98	1.04	8.5	0.03	0.688885
*MPO*	56358884	RS2243828	*G*	2.42	1.04	5.66	0.04	0.688885
*VDR*	48239835	RS1544410	*A*	0.46	0.18	1.18	0.08	0.688885
*TLR4*	120478131	RS11536889	*C*	2.38	0.74	7.62	0.08	0.688885
*ELANE*	852104	RS740021	*T*	8.12	0.69	95.29	0.09	0.688885

*Adjusted values by smoking and VPI (visible plaque index)*								
*DDX39B*	2807359	RS7766569	*G*	3.39	1.1	10.43	0.03	0.71
*MPO*	56358884	RS2243828	*G*	2.33	0.97	5.58	0.06	0.71
*ELANE*	852104	RS740021	*T*	10.9	0.88	134.6	0.06	0.71
*VDR*	48239835	RS1544410	*A*	0.47	0.19	1.18	0.09	0.71
*MMP-3*	102708787	RS650108	*A*	2.07	0.89	4.77	0.09	0.71

**Table 3:** Gene, chromosomal location, and SNPs associated with initial caries (i) unadjusted and adjusted values and *p* values and FDR *q* values.

Gene	Chr. location	Marker	Allele	OR	Lower	Upper	*p* value	FDR *q* value
*Unadjusted values*								
*VDR*	48272895	RS2228570	*T*	2.49	1.18	5.25	0.01	0.66
*MMP-3*	102708787	RS650108	*A*	2.24	1.00	5.00	0.04	0.70
*LTA*	31538244	RS2009658	G	3.78	1.18	12.1	0.04	0.70
*NCR3*	31564728	RS2736189	*T*	3.53	1.10	11.3	0.05	0.70
*DDX39B*	2807359	RS7766569	*G*	3.33	1.12	9.92	0.06	0.70

*Adjusted values by smoking and VPI (visible plaque index)*								
*VDR*	48272895	RS2228570	*T*	2.68	1.2	5.98	0.01	0.50
*LTA*	31538244	RS2009658	*G*	4.00	1.16	13.84	0.02	0.50
*MMP-3*	102708787	RS650108	*A*	2.66	1.04	6.8	0.03	0.61
*DDX39B*	2807359	RS7766569	*G*	3.07	0.95	9.92	0.05	0.67
*NCR3*	31564728	RS2736189	*T*	3.02	0.90	10.13	0.05	0.67

## References

[1] Johansson I. D., Witkowska E., Kaveh B., Lif Holgerson P., Tanner A. C. (2016). The microbiome in populations with a low and high prevalence of caries. *Journal of Dental Research*.

[2] Wang X., Shaffer J. R., Weyant R. J. (2010). Genes and their effects on dental caries may differ between primary and permanent dentitions. *Caries Research*.

[3] Allcock R. J. N., Williams J. H., Price P. (2001). The central MHC gene, BAT1, may encode a protein that down-regulates cytokine production. *Genes to Cells*.

[4] Mendonça V. R. R., Souza L. C. L., Garcia G. C. (2014). DDX39B (BAT1), TNF and IL6 gene polymorphisms and association with clinical outcomes of patients with *Plasmodium vivax* malaria. *Malaria Journal*.

[5] Price P., Wong A. M.-L., Williamson D. (2004). Polymorphisms at positions -22 and -348 in the promoter of the BAT1 gene affect transcription and the binding of nuclear factors. *Human Molecular Genetics*.

[6] Kinkade J. M., Pember S. O., Barnes K. C., Shapira R., Spitznagel J. K., Martin L. E. (1998). Differential distribution of distinct forma of myeloperoxidase in different azurophilic granule subpopulations from human neutrophils. *Biochemical and Biophysical Research Communications*.

[7] Leppilahti J. M., Hernández-Ríos P. A., Gamonal J. A. (2014). Matrix metalloproteinases and myeloperoxidase in gingival crevicular fluid provide site-specific diagnostic value for chronic periodontitis. *Journal of Clinical Periodontology*.

[8] Saari H., Suomalainen K., Lindy O., Konttinen Y. T., Sorsa T. (1990). Activation of latent human neutrophil collagenase by reactive oxygen species and serine proteases. *Biochemical and Biophysical Research Communications*.

[9] Spallarossa P., Garibaldi S., Barisione C. (2008). Postprandial serum induces apoptosis in endothelial cells: role of polymorphonuclear-derived myeloperoxidase and metalloproteinase-9 activity. *Atherosclerosis*.

[10] Sorsa T., Tjäderhane L., Konttinen Y. T. (2006). Matrix metalloproteinases: contribution to pathogenesis, diagnosis and treatment of periodontal inflammation. *Annals of Medicine*.

[11] Legrand D., Elass E., Carpentier M., Mazurier J. (2005). Lactoferrin: a modulator of immune and inflammatory responses. *Cellular and Molecular Life Sciences*.

[12] Fine D. H. (2015). Lactoferrin: a roadmap to the borderland between caries and periodontal disease. *Journal of Dental Research*.

[13] Velusamv S. K., Fine D. H., Velliyagounder K. (2014). Prophylatic effect of human lactoferrin against *Streptococcus mutans* bacteremia in lactoferrin knockout mice. *Microbes and Infection*.

[14] Heikkinen A. M., Kettunen K., Kovanen L. (2016). Inflammatory mediator polymorphisms associate with initial periodontitis in adolescents. *Clinical and Experimental Dental Research*.

[15] Benjamini Y., Hochberg Y. (1995). Controlling the false discovery rate: a practical and powerful approach to multiple testing. *Journal of the Royal Statistical Society*.

[16] Glickman M. E., Rao S. R., Schultz M. R. (2014). False discovery rate control is a recommended alternative to Bonferroni-type adjustments in health studies. *Journal of Clinical Epidemiology*.

[17] R Core Team (2017). *R: A Language and Environment for Statistical Computing, R Foundation for Statistical Computing*.

[18] Gonzalez J. R., Armengol L., Sole X. (2007). SNPassoc: an R package to perform whole genome association studies. *Bioinformatics*.

[19] Shaffer J. R., Wang X., Desensi R. S. (2012). Genetic susceptibility to dental caries on pit and fissure and smooth surfaces. *Caries Research*.

[20] Wendell S., Wang X., Brown M. (2010). Taste genes associated with dental caries. *Journal of Dental Research*.

[21] Yu M., Jiang Q. Z., Sun Z. Y., Kong Y. Y., Chen Z. (2017). Association between single nucleotide polymorphisms in vitamin D receptor gene polymorphisms and permanent tooth caries susceptibility to permanent tooth caries in Chinese adolescents. *BioMed Research International*.

[22] Izakovicova H. L., Borilova L. P., Kastovsky J. (2017). Vitamin D receptor *TaqI* gene polymorphism and dental caries in Czech children. *Caries Research*.

[23] Cogulu D., Onay H., Ozdemir Y., Aslan G. I., Ozkinay F., Eronat C. (2016). The role of vitamin D receptor polymorphisms on dental caries. *Journal of Clinical Pediatric Dentistry*.

[24] Chaussain-Miller C., Fiorett F., Goldberg M., Menashi S. (2006). The role of matrix metalloproteinases (MMPs) in human caries. *Journal of Dental Research*.

[25] Tjäderhane L., Larjava H., Sorsa T., Uitto V. J., Larmas M., Salo T. (1998). The activation and function of host matrix metalloproteinasas in dentin matrix breakdown in caries lesions. *Journal of Dental Research*.

[26] Antunes L. A., Antunes L. S., Kuchler E. C. (2016). Analysis of the association between polymorphisms in MMP2, MMP3, MMP9, MMP20, TIMP1, and TIMP2 genes with white spot lesions and early childhood caries. *International Journal of Paediatric Dentistry*.

[27] Lewis D. D., Shaffer J. R., Feingold E. (2017). Genetic association of *MMP10, MMP14*, and *MMP16* with dental caries. *International Journal of Dentistry*.

[28] Tannure P. N., Kuchler E. C., Falagan-Lotsch P. (2012). *MMP13* polymorphism decreases risk for dental caries. *Caries Research*.

[29] Karayasheva D., Glushkova M., Boteva E., Mitev V., Kadiyska T. (2016). Association study for the role of matrix metalloproteinases 2 and 3 gene polymorphisms in dental caries susceptibility. *Archives of Oral Biology*.

[30] Chapple I. L., Bouchard P., Cagetti M. G. (2017). Interaction of lifestyle, behaviour or systemic diseases with dental caries and periodontal diseases: consensus report of group 2 of the joint EFP/ORCA workshop on the boundaries between caries and periodontal diseases. *Journal of Clinical Periodontology*.

[31] Wang X., Willing M. C., Marazita M. L. (2012). Genetic and environmental factors associated with dental caries in children: the Iowa fluoride study. *Caries Research*.

[32] Benedetti G., Campus G., Strohmenger L., Lingström P. (2013). Tobacco and dental caries: a systemic review. *Acta Odontologica Scandinavica*.

[33] Tanner T., Kämppi A., Päkkilä J. (2014). Association of smoking and snuffing with dental caries occurrence in a young male population in Finland: a cross-sectional study. *Acta Odontologica Scandinavica*.

[34] Chen X., Liu Y., Yu Q. (2014). Dental caries status and oral health behaviour among civilian pilots. *Aviation, Space, and Environmental Medicine*.

